# Diagnosing helminth infections in a large reference laboratory in the United States: a 6-month pre- and post-implementation analysis of AI-augmented screening of concentrated fecal wet mounts

**DOI:** 10.1128/jcm.00165-26

**Published:** 2026-06-12

**Authors:** Cole P. Anderson, Marc Roger Couturier, Mark DeMaranville, Ryan Jensen, Madison Sant, Adam Barker, Blaine A. Mathison

**Affiliations:** 1 Department of Pathology, University of Utah 7060, Salt Lake City, Utah, USA; 2 MaineHealth NorDx, Scarborough, Maine, USA; 3 Tufts University School of Medicine 12261, Boston, Massachusetts, USA; 4 ARUP Laboratories 33294, Salt Lake City, Utah, USA; Mayo Clinic Minnesota, Rochester, Minnesota, USA

**Keywords:** ova-and-parasite, artificial intelligence, parasitology, helminth

## Abstract

Helminth infections are uncommon in the United States, yet accurate detection remains critical. Conventional ova-and-parasite examinations are labor-intensive and prone to error in low-prevalence settings. We evaluated the impact of implementing an artificial intelligence (AI)-based wet mount screening platform (WM-AI) in a high-volume reference laboratory. Detection rates, turnaround times, and specimen characteristics were compared across two 6-month periods before and after WM-AI implementation. Post-implementation, helminth detection increased from 29 to 104 patients, and client-level positivity rose from 4.5% to 12.3% (*P* < 0.0001). *Enterobius vermicularis* showed a fivefold increase in positivity (0.03% vs 0.15%, *P* < 0.0001). Median turnaround time improved from 5.2 to 4.0 days (*P* < 0.0001). Most infections were identified in the first or only specimen submitted, and over half of positive specimens contained ≤5 eggs or larvae, highlighting the challenge of detecting low-intensity infections. AI-assisted screening enhanced operational efficiency and diagnostic yield, suggesting improved sensitivity for low-burden infections. These findings support the integration of AI tools in parasitology workflows.

**IMPORTANCE:**

The data presented in our manuscript are observational after adopting a more sensitive assay in our parasitology workflow, specifically the use of artificial intelligence (AI) to pre-screen concentrated stool specimens for parasites. The notable increase in the positivity of helminth cases in our lab, which is a large reference laboratory, indicates that adopting AI as a screening tool in a parasitology laboratory can increase sensitivity of pathogenic parasites, especially helminths.

## INTRODUCTION

Parasitic diseases remain a significant global health burden, requiring sensitive laboratory diagnostics for accurate detection and management. Increased international travel and large-scale human migration have heightened the need for reliable testing in non-endemic regions, particularly as relative prevalence remains low. Despite advances in antigen detection and nucleic acid amplification tests, manual microscopy continues to be the gold standard for the identification of most intestinal parasites given its broad detection capability. However, traditional ova-and-parasite (O&P) examinations are labor-intensive, time-consuming, and highly dependent on morphological expertise. In developed countries, conventional O&P exams often yield low positivity rates (2%–5%), leading to fatigue and diagnostic errors (1). Digital imaging-based systems and artificial intelligence (AI) offer promising solutions, yet commercially available systems remain limited (2–5).

The United States is not traditionally considered endemic for most helminths. Recent surveillance studies that have focused on soil-transmitted helminths (STHs), such as hookworm and *Strongyloides*, in the southeastern United States, in areas that would normally be considered high-risk due to climate and socioeconomic factors, have shown little to no transmission of STH infections (6–9). In the United States, ascariasis is generally a zoonotic disease associated with pig farming or the use of pig feces to fertilize crops (10, 11). As such, high-volume testing in populations of low prevalence can negatively affect competency for helminth eggs and larvae detection. To address this challenge, our laboratory implemented a digital-image-based screening platform (Techcyte Fusion Parasitology Suite, Techcyte, Inc., Orem, Utah, USA) to screen for parasites in concentrated stool specimens. Our lab initially integrated Techcyte’s trichrome model in 2019, which detects common pathogenic and nonpathogenic protozoans in trichrome-stained stool specimens (12). This was expanded in 2025 to include Techcyte’s wet mount model for screening concentrated stool wet mounts for helminth eggs and larvae and protozoan cysts, oocysts, and trophozoites (13). Trends from 2019 to 2024 showed a 2%–3% increase in positivity in protozoal agents after implementation of the AI platform (unpublished data). With the addition of the wet mount portion of the AI platform (henceforth referred to as simply “WM-AI”), we expected to see an increase in positivity for helminth infections. Here, we compare the detection of helminth eggs and larvae in concentrated wet mounts of stool pre- and post-implementation of an automated scanner and analyzer system during a 1-year period.

## MATERIALS AND METHODS

### Specimen data

**Fig 1:**
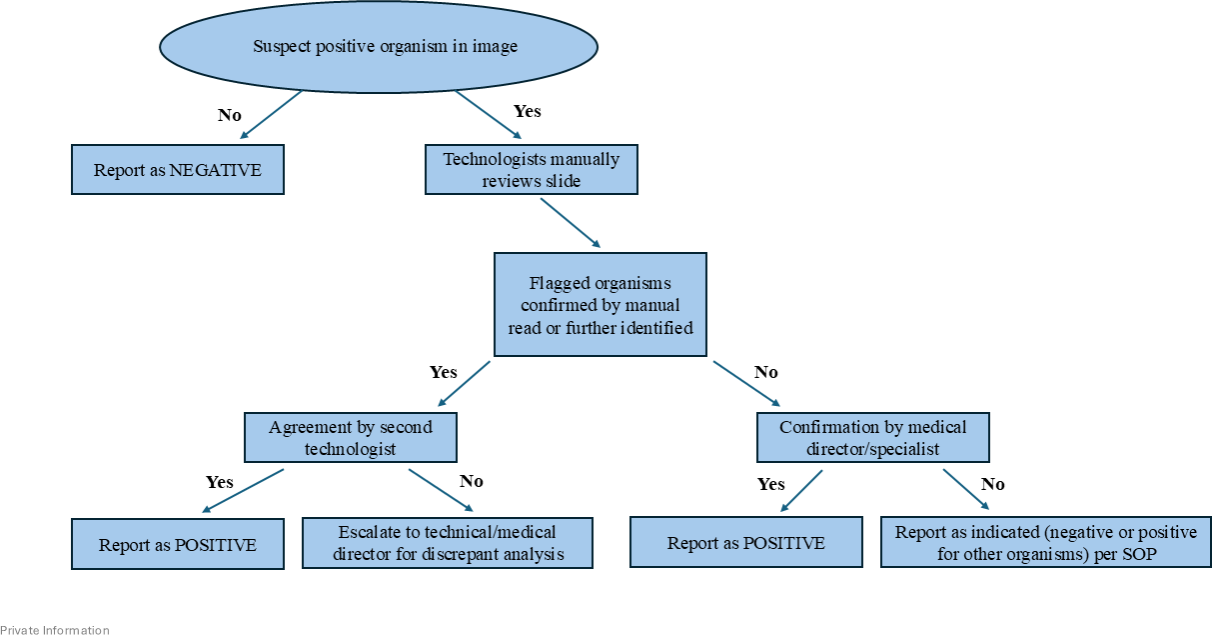
Laboratory workflow for ova-and-parasite (O&P) analysis and result verification.

Specimens submitted for O&P testing were prepared and scanned per Mathison et al. (13). Stool specimens in a variety of fixatives were concentrated using Mini Parasep SF tubes (Apacor, Wokingham, United Kingdom). Fifteen microliters of concentrated stool was mixed with 20 µL of mounting medium (1:1 10% glycerol in phosphate-buffered saline and Lugol’s iodine) and coverslipped using a 22 × 22 mm glass coverslip. When suspect parasites were detected by WM-AI, the specimen was manually reviewed by a trained technologist. After review, the specimen was backread by a second technologist. If the organisms flagged by the software could not be found by either technologist, or if there is a disagreement in the identification between the two technologists, the specimen was escalated for review by the Laboratory Director, Technical Director, or Specialist. If putative parasites detected by the software could not be detected on manual microscopy, the Laboratory or Technical Director could confirm by images only (Fig. 1) (13).

Clinical laboratory data were collected from 27 September 2024 to 26 March 2025 for the pre-WM-AI period and from 27 March 2025 to 29 September 2025 for the post-WM-AI period. Historical 3-year data were collected from 1 January 2021 to 31 December 2023 for comparison. Graphing and descriptive statistical analysis were performed using GraphPad Prism (v10). Welch’s *t*-test was used to compare the turnaround times between pre- and post-AI periods. Turnaround time was defined as the interval in days between specimen receipt to final verification. Patient ages were presented as median and interquartile range. Positivity rates between pre- and post-WM-AI study periods were compared using the N-1 Chi-squared test.

## RESULTS

### Helminth detection

**Fig 2:**
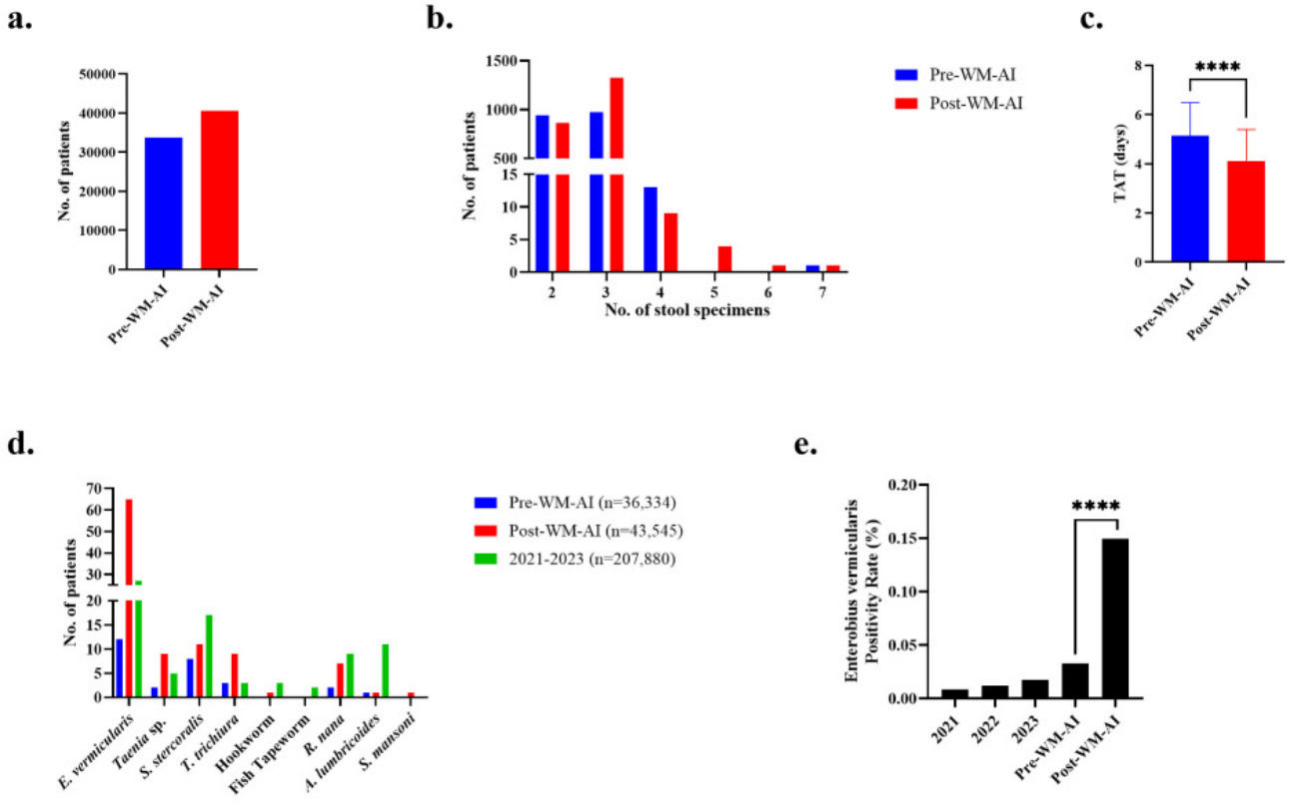
Analysis of helminth egg and larvae detection. (**a**) Number of patients where only a single stool specimen was submitted for analysis. (**b**) Number of patients where two or more stool specimens were submitted for analysis in a 7-day window. (**c**) Comparison of the mean turnaround time between pre- and post-WM-AI periods. (**d**) Comparison of the number of unique patients with parasitic helminths detected in stool specimens between pre-WM-AI, post-WM-AI, and 2021–2023 study periods. (**e**) Comparison of *Enterobius vermicularis* positivity rates from 2021 through the post-AI period. Significance stated as ****P < 0.0001.

During the 6-month pre-WM-AI period, there were 40,297 stool specimens submitted for 36,334 patients, from 507 clients with a median age of 52.7 years (interquartile range [IQR], 30.9–69.3). Single specimens were submitted for 92.8% (*n* = 33,701) of patients, while only 5.3% (*n* = 1,930) of patients had more than one specimen collected in a 7-day period (Fig. 2a and b). For the 6-month post-WM-AI period, there were 48,343 stool specimens submitted for 43,545 patients from 528 clients with a median age of 51.7 years (IQR, 31.1–68.9). Single specimens were submitted for 93.0% (*n* = 40,478) of patients, while only 5.1% (*n* = 2,204) of patients had more than one specimen collected in a 7-day period (Fig. 2a and b). Turnaround time was significantly lower during the post-WM-AI period (mean, 4.1 vs 5.1 days; *P* < 0.0001) (Fig. 2c).

Helminths were detected in 29 and 104 patients during the pre- and post-WM-AI periods, respectively (Fig. 1d). Client level positivity rate increased from 4.5% (23/507) in the pre-WM-AI period to 12.3% (65/528; *P* < 0.0001) in the post-WM-AI period. A total of 65 clients submitted specimens that were positive for helminths in the post-WM-AI period, with the top 3 accounting for 8, 5, and 5 patients, respectively. Twenty-one new clients submitted specimens for O&P testing to our institute in the post-WM-AI period, but only 3 accounted for helminth detections (*Enterobius vermicularis*, *n* = 2; *Taenia* species, *n* = 1; hookworm, *n* = 1). The most frequently detected helminths during the pre- and post-WM-AI periods were *Enterobius vermicularis* (12 vs 65), *Strongyloides stercoralis* (8 vs 11), *Taenia* species (2 vs 9), and *Trichuris trichiura* (3 vs 9). One *Taenia*-positive patient in the post-WM-AI period was not detected by AI screening due to technical issues (the scanner was inoperable over the course of a weekend and specimens were read manually) but was detected using traditional manual microscopy. Co-infection with *T. trichiura* and *Rodentolepis nana* was detected in two specimens from a single patient during the post-WM-AI period. *Ascaris lumbricoides*, *Schistosoma mansoni*, hookworm, and fish tapeworm (Diphyllobothriidae cestodes, *Diphyllobothrium*, *Dibothriocephalus*, and *Adenocephalus*) were detected only in single patients across study periods. Notably, the positivity rate for *E. vermicularis* increased fivefold (0.03% vs 0.15%, *P* < 0.0001) between the pre- and post-WM-AI study periods, where the median ages were 12.4 (IQR, 6.8–16.2) and 10.1 (IQR, 7.5–14.1) years, respectively. In contrast, annual *E. vermicularis* positivity rates from 2021 to 2023 ranged from 0.009% to 0.017% (Fig. 2e). Positivity rates for other helminths were not significantly different between study periods (Data S1). Overall, 75.9% (*n* = 101) of positive patients had only a single specimen submitted for evaluation. In the pre-WM-AI period, 93.1% (27/29) of all helminths that were detected were present in the first or only specimen examined compared to 98.1% (102/104; *P* = 0.32) in the post-WM-AI (Table 1). Helminth detections pre- and post-WM-AI periods were compared to aggregate helminth detections from 2021 to 2023 (Fig. 2d). During the 3-year period prior to the study year, helminths were detected in a total of 75 patients. The three most commonly detected helminths were *E. vermicularis* (*n* = 27), *S. stercoralis* (*n* = 17), and *A. lumbricoides* (*n* = 10). A single co-infection with hookworm and *Ascaris* was detected. Similar to the pre- and post-WM-AI periods, 92.1% (70/76) of all helminths that were detected were present in the first or only specimen examined (Table 1). During the pre-WM-AI period, two patients were negative on initial specimens, but were positive (*R. nana* and *S. stercoralis*) on subsequent specimens collected within a seven-day period. Similarly, during the post-WM-AI period, two patients were negative on initial specimens but were positive (*E. vermicularis* and *S. stercoralis*) on subsequent specimens collected within a 7-day period. *S. stercoralis* was detected in one patient during the post-WM-AI period 4 months after their initial specimen was negative (data not shown).

**TABLE 1:** Helminths detected by stool ova-and-parasite (O&P) examination pre- and post-AI wet mount implementation

	No. of unique patients with at least one positive helminth specimen by ova-and-parasite (O&P) exam		
Parasite	6-month pre-WM-AI^*a*^ (total)^*b*^	6-month Post-WM-AI (total)	2021–2023 (total)
*Taenia* species	2 (2)	9 (9^*d*^)	4 (5)
*Enterobius vermicularis*	12 (12)	64 (65)	26 (27)
*Strongyloides stercoralis*	7 (8)	10 (11)	14 (17)
*Trichuris trichiura*	3 (3)	9 (9)	3 (3)
*Rodentolepis nana*	1 (2)	7 (7)	8 (9)
*Ascaris lumbricoides*	1 (1)	1 (1)	10 (10)
*Schistosoma mansoni*	0 (0)	1 (1)	0 (0)
Hookworm	0 (0)	1 (1)	3 (3)
Fish tapeworm^*c*^	0 (0)	0 (0)	2 (2)

^*a*^ Number of patients with corresponding parasite detected in a single specimen or first specimen when more than one specimen was submitted.

^*b*^ The total number in parenthesis reflects the number of unique patients that were positive for the relevant parasite, where an initial specimen was negative but positive on a subsequent specimen collected within a 7-day period.. If a parasite was detected in multiple specimens within a series, it was counted only once toward the total.

^*c*^ Fish tapeworm category includes cestodes in the genera *Dibothriocephalus*, *Diphyllobothrium*, and *Adenocephalus*.

^*d*^ One patient was detected by manual microscopy due to technical issues.

Helminth co-infections with at least one protozoan were observed in 17.2% (*n* = 5) and 22.1% (*n* = 23) of patients in the pre- and post-WM-AI periods, respectively (Data S2). Of the protozoan co-infections, *Blastocystis* species and *Entamoeba coli* were the two most commonly observed (Data S2).

**Fig 3:**
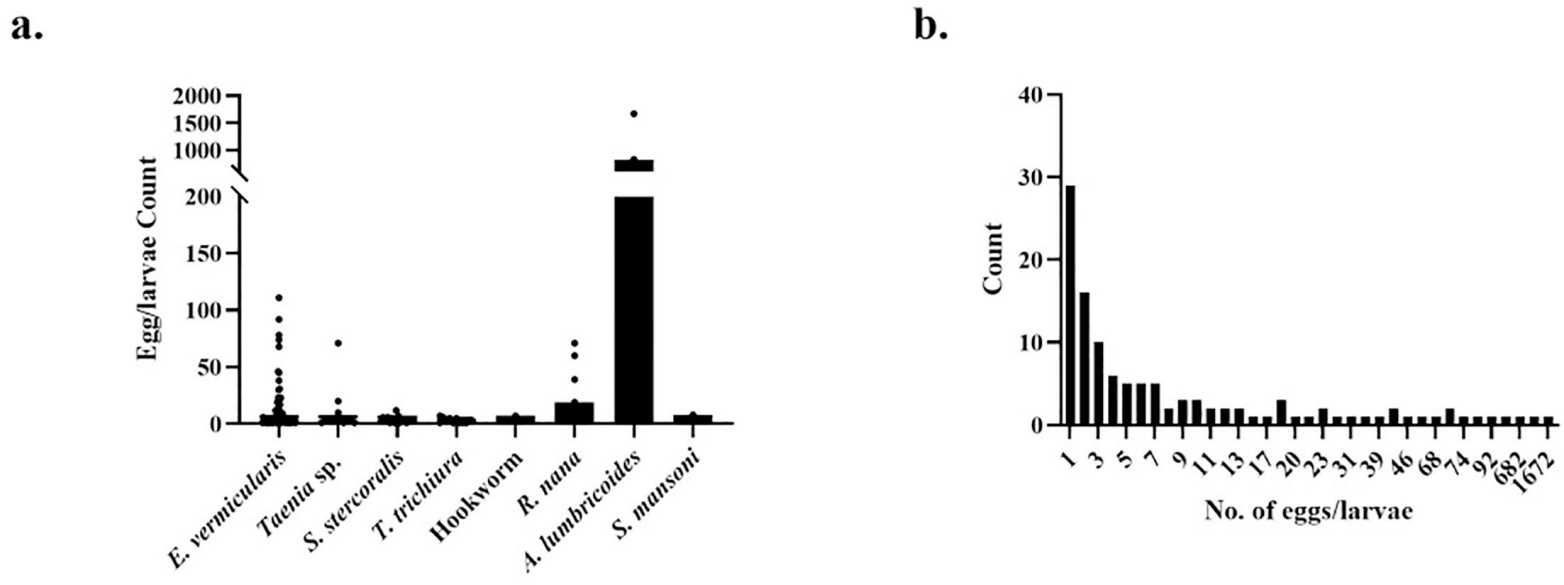
Analysis of egg and larvae counts. (**a**) Enumeration of helminth eggs or larvae detected by wet-mount AI. Bars represent the median count. (**b**) Frequency distribution of eggs or larvae counts detected by wet-mount AI.

**Fig 4:**
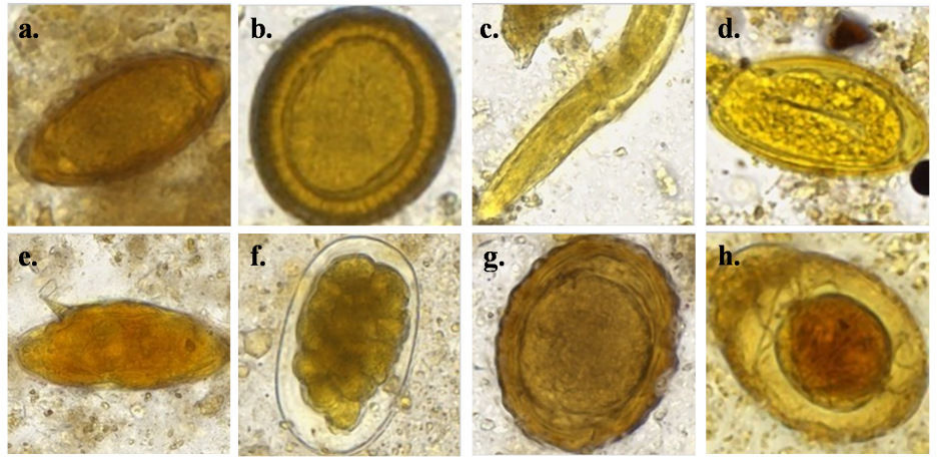
Representative images of helminth eggs or larvae captured by the AI software, (**a**) *Trichuris trichiura*, (**b**) *Taenia* sp., (**c**) *Strongyloides stercoralis*, (**d**) *Enterobius vermicularis*, (**e**) *Schistosoma mansoni*, (**f**) hookworm, (**g**) *Ascaris lumbricoides*, and (**h**) *Rodentolepis nana*.

Median counts for eggs and larvae flagged by the software ranged from 2 (*T. trichiura*) to 828 (*A. lumbricoides*) (Fig. 3a). The highest egg burden was observed in a single patient positive for *A. lumbricoides*. In this patient, three serially collected stool specimens contained 1672, 828, and 682 eggs. Of note, 56.4% (*n* = 66) of specimens had ≤5 eggs/larvae detected in the specimen, and only a single egg or larva was detected in 24.8% (*n* = 29) of specimens (Fig. 3b). Less than 15 eggs or larvae were observed in specimens positive for *S. stercoralis*, *T. trichiura*, hookworm, and *S. mansoni*. Software flagged images are confirmed by manual microscopy of a freshly prepared wet mount of the stool specimen. In nine specimens, results were verified by the technical director by image analysis alone because eggs or larvae could not be identified by manual microscopy backreading or discrepant analysis (see Data S3 for images). This occurred in specimens where only a single egg or larva of *T. trichiura* (*n* = 4), *S. stercoralis* (*n* = 1), and *E. vermicularis* (*n* = 4) was identified by the AI software. Representative images of helminth eggs and larvae captured by the AI software are illustrated in Fig. 4.

## DISCUSSION

The implementation of AI-assisted screening for helminth detection in stool wet mounts improved diagnostic yield and operational efficiency in our high-volume reference laboratory. Most notably, the positivity rate for *E. vermicularis* increased fivefold compared to the pre-AI period and was markedly higher than historical rates from 2021 to 2023 (see below). This suggests that AI screening enhances detection of low-intensity infections, which are often missed during manual microscopy.

There are numerous reasons helminths may be missed during manual microscopy. One could be the intermittent and often light shedding of eggs by some species. Another could be due to reader error. Despite rigorous training, quarterly competency, and periodic proficiency testing, there are several possible reasons for such discrepancies. When fully staffed, our laboratory has 22 technologists (including float technologists that rotate through different labs) trained to read O&P exams. Despite standardizations in training, once technologists are reading on their own, they are likely to develop their own tendencies which are inherently difficult to track and monitor. For example, wet mount preps could be made with slight variations in volume, and different people will read specimens at different rates. Also, organisms less commonly seen in the lab, including helminths, may be more likely to be overlooked due to unfamiliarity. One of the benefits of AI screening is that it helps remove or reduce many of those factors, allowing the technologist to scrutinize specimens with a higher likelihood of being positive.

A single stool specimen was submitted for evaluation for most patients during the study period. Of the positive patients, nearly all were positive in their first or only specimen evaluated. The first-or-only specimen yield increased from 93.1% pre-WM-AI to 97.1% post-WM-AI. These high detection rates in single specimens raise the often-debated question regarding the need for serial stool O&P examinations. Authoritative microbiology manuals and published guidelines continue to recommend multiple stool specimens collected over 5 to 7 days despite a lack of consensus on the matter. Previous studies have shown high sensitivity with a single stool examination. One study demonstrated that over 90% of parasites were detected in the first specimen submitted, and when prevalence is low, negative predictive value exceeded 98% (14). Another found that the sensitivity of a single stool examination for hookworm, *T. trichiura*, and *A. lumbricoides* was 77.4%, 88.8%, and 91.6%, respectively (15). While helminths are often shed more intermittently than protozoans, further studies are warranted to determine if sensitivity increases using AI can eliminate the need for multiple O&P collections.

A key observation was that most positive specimens contained very few eggs or larvae—over half had ≤5, and nearly one-quarter had only a single egg or larva. This highlights the challenge of detecting low-burden infections. Studies in high-endemic areas have shown that traditional methods like double-slide Kato-Katz have poor sensitivity in low-intensity infections, and molecular assays such as quantitative PCR outperform microscopy in these settings (16–18). While studies are limited, implementation of AI screening for STH infections in low-resource settings with high prevalence has shown promise. Using a deep learning system of digitized Kato-Katz smears in Kenya, Lundin et al. (19) observed a 10% increase in sensitivity in detecting light-intensity STH infections compared to manual microscopy.

One of the most interesting outcomes of this study was the increase in specimens positive for *E. vermicularis*. Pinworm is well-adapted to the human host. Infections occur worldwide, and the direct route of infection means a greater percentage of the global population is at risk of disease, and cases are not necessarily more common in resource-poor regions or areas where poor infrastructure allows soil, food, and water to be contaminated with human feces. Classic symptoms include nocturnal perianal pruritis, which can lead to sleep disturbances, childhood enuresis, and ulceration, anal dermatitis, perianal folliculitis, or ischiorectal abscesses from scratching (20). Still, roughly one-third of cases are asymptomatic (21). When pinworm infection is suspected, the primary diagnostic methods include the cellulose tape test or the use of specialized commercial paddles. O&P exam is not considered a helpful diagnostic tool since the eggs are shed outside the lumen of the intestine (20). The large increase in pinworm detection in our lab since the implementation of WM-AI raises two questions. First, are clinicians suspecting pinworm infection but ordering a less sensitive assay? If this is the case, the increased detection using WM-AI represents a clear advantage in disease detection. Second, are patients presenting with symptoms more consistent with requiring an O&P exam (e.g., diarrhea lasting longer than 7 days), and they just happen to be infected with pinworm as well? Given how well adapted *E. vermicularis* is to the human host and the high rate of asymptomatic infections, it is possible that a larger than expected percentage of the population may be harboring pinworm. It should be noted that pinworm detection by O&P is likely heavily biased by the time of day the stool was collected. Since eggs are deposited by females in the perianal area at night, the greatest chance of detection is likely to be the first bowel movement of the day, preferably before bathing. In the 65 patients positive for *E. vermicularis* in the post-WM-AI period, 13 were also positive for protozoans, and 10 of those had protozoal agents that have been implicated as potentially causing diarrhea (*Dientamoeba fragilis* and *Blastocystis* sp.) and thus prompt O&P testing.

Five patients in the post-WM-AI period (one in the pre-WM-AI period) were co-infected with *E. vermicularis* and *D. fragilis*. In 1956, it was proposed that *E. vermicularis* may serve as a vector for *D. fragilis* (22). This was based partially on the similarities to the poultry trichomonad *Histomonas meleagridis*, which is carried on the eggs of the nematode *Heterakis gallinarum*. That theory has generally fallen out of favor, and the association is more likely due to these two organisms having a direct fecal-oral route. Given how frequently *D. fragilis* is reported in our laboratory, one would expect more co-infections given the enhanced diagnosis using AI if that was a true biological association. During the 6-month period before the implementation of AI, 156 patients were reported to have *D. fragilis* but not *E. vermicularis*, while in the 6-month period post-AI, 126 patients were reported to have *D. fragilis* infection without *E. vermicularis*. Co-infections with protozoa were observed in approximately 20% of helminth-positive cases, highlighting the importance of comprehensive parasitological evaluation.

While the observation of such an increase in pinworm-positive specimens was unexpected, the authors are not making any claims or suggestions that O&P testing should supplant the traditional testing of cellulose tape prep or specialized paddles at this time. Pinworm paddle volume and positivity rate between study periods was not significantly different at our institution. Positivity rates were 8.0 (8/99) and 11.0% (10/93) during the pre- and post-WM-AI periods, respectively. Parallel pinworm paddle and O&P evaluation was performed for 22 patients across both study periods, and only a single patient was positive on both.

As this was not a controlled study, but rather an observation after adapting a more sensitive technology into the O&P processes, there are several limitations. The data were all retrospective, without clinical or travel history, thus limiting epidemiological interpretation. Additionally, a head-to-head comparison between blinded technologists and AI would have provided a more rigorous assessment of analytical performance. Extending the study period beyond six months and incorporating prospective data could strengthen conclusions.

In summary, the use of WM-AI in a large reference lab setting in the United States allowed for increased detection of helminths compared to the immediate period before WM-AI implementation. The most significant increase was seen for *Enterobius vermicularis*, which could represent an unintended and unexpected reduction in diagnostic gap for this prevalent, cosmopolitan helminth. The implementation of AI in the clinical parasitology laboratory could increase detection rates for helminths and other parasites observed less frequently or less familiar to the reading technologists.

## SUPPLEMENTAL MATERIAL

Supplemental Data jcm.00165-26-s0001.xlsx Data S1 and S2.

Data S3 jcm.00165-26-s0002.docx Images of single eggs and larvae detected by AI but not confirmed on manual read.

ASM does not own the copyrights to Supplemental Material that may be linked to, or accessed through, an article. The authors have granted ASM a non-exclusive, world-wide license to publish the Supplemental Material files. Please contact the corresponding author directly for reuse.

## References

[1] George E. 2010. Occupational hazard for pathologists. Am J Clin Pathol 134:543–548. doi:10.1309/AJCPI2Y0HCKGLWWP 20231606

[2] Ghazali KH, Hadi RS, Mohamed Z. 2013. Automated system for diagnosis intestinal parasites by computerized image analysis. MAS 7:98–114. doi:10.5539/mas.v7n5p98

[3] Yang YS, Park DK, Kim HC, Choi MH, Chai JY. 2001. Automatic identification of human helminth eggs on microscopic fecal specimens using digital image processing and an artificial neural network. IEEE Trans Biomed Eng 48:718–730. doi:10.1109/10.923789 11396601

[4] Intra J, Taverna E, Sala MR, Falbo R, Cappellini F, Brambilla P. 2016. Detection of intestinal parasites by use of the cuvette-based automated microscopy analyser sediMAX. Clin Microbiol Infect 22:279–284. doi:10.1016/j.cmi.2015.11.014 26679923

[5] Boonyong S, Hunnangkul S, Vijit S, Wattano S, Tantayapirak P, Loymek S, Wongkamchai S. 2024. High-throughput detection of parasites and ova in stool using the fully automatic digital feces analyzer, orienter model fa280. Parasit Vectors 17:13. doi:10.1186/s13071-023-06108-1 38185634 PMC10771706

[6] Bleakley H. 2007. Disease and development: evidence from hookworm eradication in the American South. Q J Econ 122:73–117. doi:10.1162/qjec.121.1.73 24146438 PMC3800113

[7] McKenna ML, McAtee S, Bryan PE, Jeun R, Ward T, Kraus J, Bottazzi ME, Hotez PJ, Flowers CC, Mejia R. 2017. Human intestinal parasite burden and poor sanitation in Rural Alabama. Am J Trop Med Hyg 97:1623–1628. doi:10.4269/ajtmh.17-0396 29016326 PMC5817782

[8] Bradbury RS, Lane M, Arguello I, Handali S, Cooley G, Pilotte N, Williams JM, Jameson S, Montgomery SP, Hellmann K, Tharp M, Haynie L, Galloway R, Brackin B, Kirmse B, Stempak L, Byers P, Williams S, Faruque F, Hobbs CV. 2021. Parasitic disease Surveillance, Mississippi, USA. Emerg Infect Dis 27:2201–2204. doi:10.3201/eid2708.204318 34287125 PMC8314824

[9] Bradbury RS, Martin L, Malloch L, Martin M, Williams JM, Patterson K, Sanders C, Singh G, Arguello I, Rodriguez E, Byers P, Haynie L, Qvarnstrom Y, Hobbs CV. 2023. Surveillance for soil-transmitted helminths in high-risk County, Mississippi, USA. Emerg Infect Dis 29:2533–2537. doi:10.3201/eid2912.230709 37987591 PMC10683803

[10] Miller LA, Colby K, Manning SE, Hoenig D, McEvoy E, Montgomery S, Mathison B, de Almeida M, Bishop H, Dasilva A, Sears S. 2015. Ascariasis in humans and pigs on small-scale farms, Maine, USA, 2010–2013. Emerg Infect Dis 21:332–334. doi:10.3201/eid2102.140048 25626125 PMC4313629

[11] Mathison B.A, Pritt BS. 2018. A systematic overview of zoonotic helminth infections in North America. Lab Med 49:e61–e93. doi:10.1093/labmed/lmy029 30032297

[12] Mathison BA, Kohan JL, Walker JF, Smith RB, Ardon O, Couturier MR. 2020. Detection of intestinal protozoa in trichrome-stained stool specimens by use of a deep convolutional neural network. J Clin Microbiol 58:e02053-19. doi:10.1128/JCM.02053-19 32295888 PMC7269375

[13] Mathison BA, Knight K, Potts J, Black B, Walker JF, Markow F, Wood A, Bess D, Dixon K, Cahoon B, Hymas W, Couturier MR. 2025. Detection of protozoan and helminth parasites in concentrated wet mounts of stool using a deep convolutional neural network. J Clin Microbiol 63:e0106225. doi:10.1128/jcm.01062-25 41117595 PMC12607898

[14] Branda JA, Lin TYD, Rosenberg ES, Halpern EF, Ferraro MJ. 2006. A rational approach to the stool ova and parasite examination. Clin Infect Dis 42:972–978. doi:10.1086/500937 16511762

[15] Marti H, Koella JC. 1993. Multiple stool examinations for ova and parasites and rate of false-negative results. J Clin Microbiol 31:3044–3045. doi:10.1128/jcm.31.11.3044-3045.1993 8263196 PMC266208

[16] Dunn JC, Papaiakovou M, Han KT, Chooneea D, Bettis AA, Wyine NY, Lwin AMM, Maung NS, Misra R, Littlewood DTJ, Anderson RM. 2020. The increased sensitivity of qPCR in comparison to Kato-Katz is required for the accurate assessment of the prevalence of soil-transmitted helminth infection in settings that have received multiple rounds of mass drug administration. Parasit Vectors 13:324. doi:10.1186/s13071-020-04197-w 32580759 PMC7315547

[17] Benjamin-Chung J, Pilotte N, Ercumen A, Grant JR, Maasch JRMA, Gonzalez AM, Ester AC, Arnold BF, Rahman M, Haque R, Hubbard AE, Luby SP, Williams SA, Colford JM. 2020. Comparison of multi-parallel qPCR and double-slide Kato-Katz for detection of soil-transmitted helminth infection among children in rural Bangladesh. PLoS Negl Trop Dis 14:e0008087. doi:10.1371/journal.pntd.0008087 32330127 PMC7202662

[18] Nikolay B, Brooker SJ, Pullan RL. 2014. Sensitivity of diagnostic tests for human soil-transmitted helminth infections: a meta-analysis in the absence of a true gold standard. Int J Parasitol 44:765–774. doi:10.1016/j.ijpara.2014.05.009 24992655 PMC4186778

[19] Lundin J, Suutala A, Holmström O, Henriksson S, Valkamo S, Kaingu H, Kinyua F, Muinde M, Lundin M, Diwan V, Mårtensson A, Linder N. 2024. Diagnosis of soil-transmitted helminth infections with digital mobile microscopy and artificial intelligence in a resource-limited setting. PLoS Negl Trop Dis 18:e0012041. doi:10.1371/journal.pntd.0012041 38602896 PMC11008773

[20] Wendt S, Trawinski H, Schubert S, Rodloff AC, Mössner J, Lübbert C. 2019. The diagnosis and treatment of pinworm infection. Dtsch Arztebl Int 116:213–219. doi:10.3238/arztebl.2019.0213 31064642 PMC6522669

[21] Friesen J, Bergmann C, Neuber R, Fuhrmann J, Wenzel T, Durst A, Müller M, Ignatius R. 2019. Detection of Enterobius vermicularis in greater Berlin, 2007–2017: seasonality and increased frequency of detection. Eur J Clin Microbiol Infect Dis 38:719–723. doi:10.1007/s10096-019-03495-1 30712227

[22] Burrows RB, Swerdlow MA. 1956. Enterobius vermicularis as a probable vector of Dientamoeba fragilis. Am J Trop Med Hyg 5:258–265. doi:10.4269/ajtmh.1956.5.258 13302621

