# Supplementary material for: Diagnosing helminth infections in a large reference laboratory in the United States: a 6-month pre- and post-implementation analysis of AI-augmented screening of concentrated fecal wet mounts
Source: J Clin Microbiol. 2026 Jun 12;64(7):e00165-26. doi: 10.1128/jcm.00165-26 (PMC13343833; doi:10.1128/jcm.00165-26)
Supplement: Data S3 — Images of single eggs and larvae detected by AI but not confirmed on manual read. [file jcm.00165-26-s0002.docx]

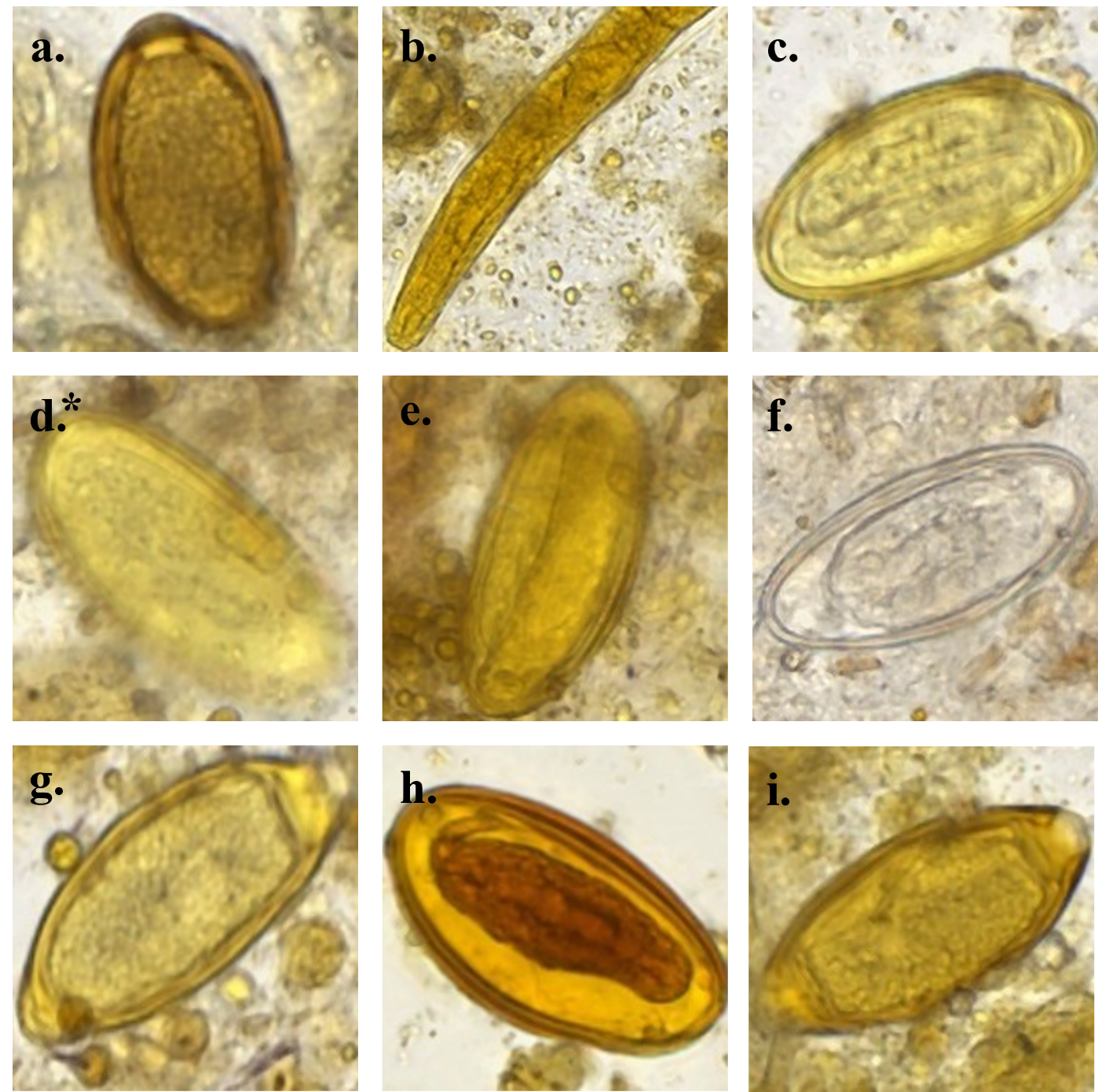


Figure S1. Techcyte images for specimens where only a single egg or larva was imaged but could not be found by a minimum of two readers after manual evaluation. (a.) *Trichuris trichiura*, (b.) *Strongyloides stercoralis*., (c.) *Enterobius vermicularis*, (d.) *E. vermicularis*, mis-identified as *Trichuris* by the software (class confusion), (e.) *E. vermicularis*, (f.) *Enterobius vermicularis*, (g.) *T. trichiura*, (h.) *Enterobius vermicularis*, (i.) *T. trichiura.*
